# Assessment of variability in pulmonary fissures using multidetector computed tomography: a short review

**DOI:** 10.1007/s00117-025-01436-y

**Published:** 2025-04-11

**Authors:** Berin Tuğtağ Demir, Engin Çiftçioğlu, Fatih Çankal

**Affiliations:** 1 Faculty of Medicine, Department of Anatomy, Ankara Medipol University, Ankara, Turkey; 2 Faculty of Medicine, Department of Anatomy, ondokuz mayıs university, Samsun, Turkey; 3 Faculty of Medicine, Department of Radiology, Pursaklar Hospital, Ankara, Turkey

**Keywords:** Accessory lung fissures, Pulmonary lobe variations, Computed tomography, Pulmonary lobes, Radiologic anatomy, Akzessorische Lungenfissuren, Lungenlappenvarianten, Computertomographie, Lungenlappen, Radiologische Anatomie

## Abstract

**Background:**

This study investigated the variability in pulmonary fissures, focusing on their presence, absence, or incompleteness, and how these variations contribute to the formation of accessory lobes.

**Objective:**

Using multidetector computed tomography (MDCT), the study aimed to define lung morphology in terms of major, minor, and accessory fissures.

**Material and methods:**

A descriptive analysis was conducted of MDCT images from 576 lungs (288 individuals). The study group comprised 162 male (56.3%) and 126 female (43.8%) patients.

**Results:**

In the right lung, 35.1% of cases exhibited an incomplete horizontal fissure, while in the left lung, accessory horizontal fissures were complete in 8.3% and incomplete in 10.2% of cases. Accessory fissures were present in 81.59% of right lungs and 47.22% of left lungs. The most common accessory fissures were located between the medial basal-anterior basal segments (44.4%), superior and basal segments (19.4%), and anterior basal-lateral basal segments (19.4%) of the lower lobe. No significant gender or lung-side differences were noted in the occurrence of fissures (*p* > 0.05).

**Conclusion:**

The study revealed significant variability in the frequency of major, minor, and accessory pulmonary fissures. Understanding these variations is crucial in shedding light on unusual clinical presentations in lung pathologies and in facilitating an accurate diagnosis and surgical planning.

## Abstract (de)

**Hintergrund:**

In der vorliegenden Studie wurde die Variabilität von Lungenfissuren untersucht, der Fokus lag dabei auf deren Vorhandensein, Fehlen oder unvollständigem Vorliegen und darauf, wie diese Varianten zur Bildung von akzessorischen Lungenlappen beitragen.

**Ziel:**

Unter Einsatz der Multidetektor-Computertomographie (MDCT) zielte die vorliegende Studie darauf ab, die Lungenmorphologie in Bezug auf Haupt‑, Neben- und akzessorische Fissuren zu definieren.

**Material und Methoden:**

Dazu wurde eine deskriptive Auswertung von MDCT-Aufnahmen von 576 Lungen (288 Personen) durchgeführt. In der Studiengruppe waren 162 männliche (56,3 %) und 126 weibliche (43,8 %) Teilnehmer.

**Ergebnisse:**

In der rechten Lunge fand sich in 35,1 % der Fälle eine unvollständige horizontale Fissur, während es in der linken Lunge in 8,3 % vollständige und in 10,2 % der Fälle unvollständige akzessorische horizontale Fissuren gab. Akzessorische Fissuren lagen in 81,59 % der rechten Lungen und in 47,22 % der linken Lungen vor. Die häufigsten akzessorischen Fissuren waren zwischen dem medialen basalen und dem anterioren basalen Segment (44,4 %), dem superioren und dem basalen Segment (19,4 %) sowie dem anterioren basalen und dem lateralen basalen Segment (19,4 %) des Unterlappens lokalisiert. Es wurden keine signifikanten Geschlechts- oder Lungenseitenunterschiede beim Auftreten der Fissuren festgestellt (*p* > 0,05).

**Schlussfolgerung:**

In der Studie ergab sich eine signifikante Variabilität bei der Häufigkeit von Haupt‑, Neben- und akzessorischen Lungenfissuren. Das Verständnis dieser Varianten ist entscheidend, um ungewöhnliche klinische Manifestationen von Lungenerkrankungen zu erhellen und eine genaue Diagnose und chirurgische Planung zu erleichtern.

## Introduction

The lungs are partitioned into lobes by a dual-layered infolding of the visceral pleura, known as *fissures* [1]. These pulmonary interlobar fissures serve as pivotal landmarks in pulmonary anatomy, comprising a double-membrane structure formed by the invagination of the visceral pleura. Typically measuring 1–3 mm in thickness, these fissures include the right oblique fissure, horizontal fissure, and left oblique fissure [2, 3]. Pulmonary fissures may be complete, incomplete, or absent. Oblique fissures extend from the lung surface to the hilum, separating the organ into distinct upper and lower lobes, interconnected solely by the lobar bronchi and vessels. In the right lung, a horizontal fissure extends from the anterior margin to the oblique fissure, demarcating a wedge-shaped middle lobe from the upper lobe [2].

During fetal development, after the seventh week, lung buds proliferate within the mesothelium-lined pleural cavity. The mesothelium lining eventually evolves into the visceral and parietal pleural surfaces. The visceral pleura projects into the interlobar spaces between each developing lobar bronchus, forming pulmonary fissures, thereby enabling independent expansion and contraction of each lobe [4]. In some instances, an incomplete visceral pleural covering results in incomplete fissures, leading to continuity between adjacent lobes. Such incomplete fissures can modify the typical collapse patterns observed in patients with endobronchial lesions. An incomplete major fissure may induce an abnormal fluid appearance in cases of pleural effusion. While accessory fissures are relatively common, their detection in radiographs and computed tomography (CT) scans can be challenging. Accurate identification of pulmonary interlobar fissures and their variations is essential for pinpointing the location of pulmonary lesions, assessing disease progression, selecting surgical interventions, and implementing endoscopic therapy [5, 6].

An accessory fissure is defined as a cleft of varying depth, lined by visceral pleura, usually occurring between bronchopulmonary segments [7]. Numerous accessory fissures have been documented by anatomists and radiologists [8, 9]. A comprehensive understanding of these fissures is instrumental in evaluating the extent of pulmonary diseases, localizing segmental pulmonary lesions, and assisting in the differential diagnosis of accessory fissures from standard anatomical and pathological structures [10].

This study focused on the investigation and analysis of pulmonary interlobar fissures, their variations, and accessory fissures in the Turkish population, using multidetector CT (MDCT) images. The objective was to delineate the morphological differences in lung fissures and lobes among this population, comparing the findings with prior studies. Such insights could yield novel data on lung lobes and fissures, thereby facilitating radiologists and surgeons in making precise diagnoses and planning surgeries effectively.

## Material and methods

### Patients

This retrospective cross-sectional study reviewed the MDCT scans of 288 patients (162 male, 126 female patients) performed between June 2016 and September 2021. This retrospective cross-sectional study was conducted after approval was obtained from the clinical research ethics committee of Ondokuz Mayis University (OMUKAEK NO: 2021/494). The patients, ranging in age from 18 to 82 years with a mean age of 44.95 years, were referred for various indications. Patients with pulmonary lesions that distorted or obliterated the pulmonary parenchyma, those with pleural disease, and those who had undergone prior thoracic surgery were excluded from the study. Power analysis was performed using the G*Power (v3.1.9.7) program to determine the number of samples. The power of the study is expressed as 1‑β (β = type II error probability). In the calculation, the effect size (*d*) to obtain 80% power at α = 0.05 was found to be 0.46. Accordingly, the research was conducted with a total of 285 people.

### Imaging procedure

**Fig. 1:**
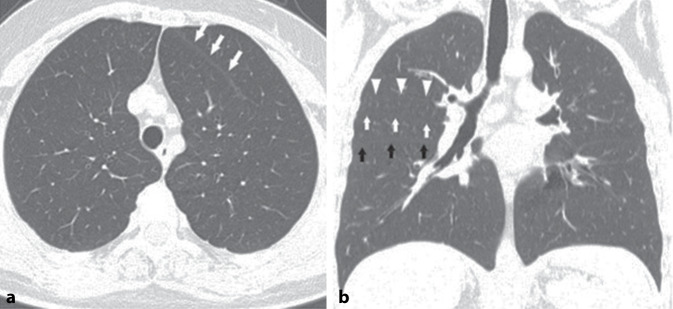
**a** Accessory horizontal fissure on the left (white arrows indicate the fissure; transverse CT image). **b** Accessory fissure between the anterior and posterior segments of the upper lobe on the right (white arrows show the horizontal fissure, black arrows show the oblique fissure, white arrowheads indicate the accessory fissure; coronal CT image)

**Fig. 2:**
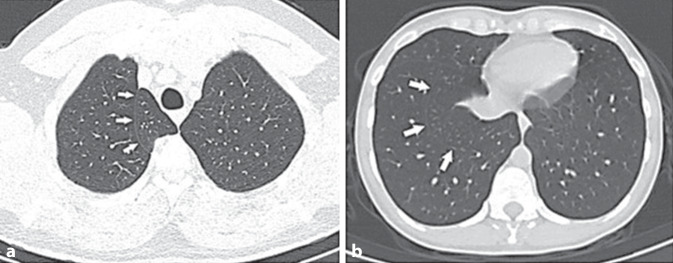
**a** Accessory azygos fissure on the right (white arrows indicate the fissure; transverse CT image). **b** Accessory fissure between the medial basal segment and anterior basal segment of the lower lobe on the right (white arrows indicate the fissure; transverse CT image)

The CT scans were performed using a General Electric IQ™ 32-slice spiral scanner (Cincinnati, OH 45215). Scans were taken in suspended inspiration, covering the area from the lung apex to the diaphragm. Parameters included 1.25-mm-thin sections, a tube voltage of 120–130 kV, and a tube current of 200–360 mAs. Images were evaluated by one radiologist (also an anatomist) and two anatomists, with final decisions reached by consensus. The evaluation focused on the lung structures of patients, investigating the presence of all segments. Fissure segmentation followed the methodology of Lange and Walsh [11]. The aim was to delineate the boundaries of all lobes and segments in the CT examinations. All potential intersegmental and intrasegmental pleural folds were assessed as fissures and documented. Fissures extending from the pulmonary hilum to the costal pleura were classified as complete. Those originating from the periphery or hilum but not fully covering the parenchyma were deemed incomplete and categorized based on whether they caused less or more than half of the segmentation/separation in the parenchyma (Figs. 1 and 2).

### Statistical analysis

Descriptive statistics were employed to characterize the occurrence of incomplete and accessory fissures. Fisher’s exact test and Yates’s chi-square test were utilized to evaluate potential differences between gender and lung side and to explore correlations between the presence of incomplete and accessory fissures and atypical localizations of interlobar fissures. Additionally, McNemar’s test of symmetry was applied to compare the right and left lungs. Data analysis was performed using SPSS software, version 22. All statistical tests were conducted with a significance level set at 0.05.

## Results

### Major fissures

**Fig. 3:**
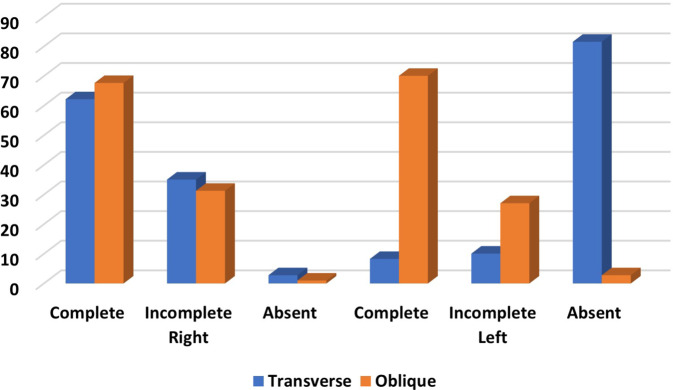
Summary of variations in horizontal and oblique fissures in the left and right lungs

The study encompassed 162 male (56.3%) and 126 female (43.8%) patients. In the right lung, 62.2% of cases exhibited a complete horizontal fissure, while 35.1% had an incomplete one. On the left side, accessory horizontal fissures were complete in 8.3% and incomplete in 10.2% of cases. Oblique fissures were predominantly complete in both the right (67.6%) and left (70.1%) lungs (Fig. 3).

Regarding horizontal fissures, in the right lung, they were complete in 62.2% and incomplete in 35.1% of cases, while in the left lung, they were complete in 8.3% and incomplete in 10.1% of cases. Oblique fissures were complete in 67.7% and incomplete in 31.3% of cases in the right lung, and in the left lung they were complete in 70.1% and incomplete in 27.1% of cases.

In both genders, the right lung predominantly exhibited a complete horizontal fissure type (male: 66.7%, female: 56.3%), followed by incomplete (male: 30.9%, female: 40.5%) and absent (male: 2.5%, female: 3.2%) types. In the left lung, horizontal fissures were notably absent in about 80% of cases in both male and female patients. No significant gender differences were observed in the variations in horizontal fissures (*p* > 0.05). Oblique fissures were most frequently complete in both male and female patients on both sides. No significant gender differences were found in the variations in the horizontal fissure type (Table 1).

**Table 1:** Distribution of fissural variations

		Complete		Incomplete		Absent	
		Female, *n* (%)	Male, *n* (%)	Female, *n* (%)	Male, *n* (%)	Female, *n* (%)	Male, *n* (%)
Horizontal	Right	71 (56.3)	108 (66.7)	51 (40.5)	50 (30.9)	4 (3.2)	4 (2.5)
	Left (accessory)	7 (5.6)	17 (10.5)	15 (11.9)	14 (8.6)	104 (82.5)	131 (80.9)
Oblique	Right	88 (69.8)	107 (66.0)	35 (27.8)	55 (34.0)	3 (2.4)	0 (0)
	Left	90 (71.4)	112 (69.1)	33 (26.2)	45 (27.8)	3 (2.4)	5 (3.1)

### Accessory fissures

**Fig. 4:**
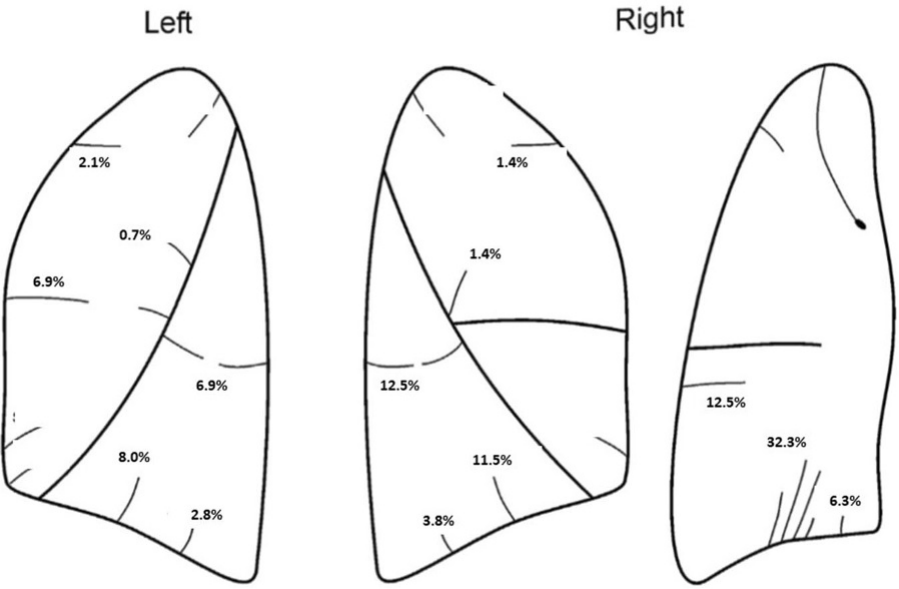
Localization of some accessory fissures and their frequency (%)

In the right lung, the three most prevalent accessory fissure variations were identified between the mediobasal and anterobasal segments of the lower lobe (32.3%), the superior and basal segments (12.5%), and the anterobasal and laterobasal segments (11.5%). In the left lung, the most common accessory fissure variations occurred between the mediobasal and anterobasal segments (12.2%), the mediobasal and posterobasal segments (8.3%), and the superior and basal segments (6.9%; Table 2; Fig. 4). Accessory fissures were present in 81.59% of right lungs and 47.22% of left lungs.

**Table 2:** Incidence of variation in accessory fissures in right and left lung

Accessory fissures	Total, *n *(%)	Right lung, *n *(%)	Left lung, *n *(%)
Azygos	5 (1.7)	5 (1.7)	–
Apical with posterior (superior)	15 (5.2)	9 (3.1)	6 (2.1)
Between superior and basal (inferior) superior oblique fissure	56 (19.4)	36 (12.5)	20 (6.9)
Medial basal boundary	0 (0)	0 (0)	0 (0)
Apical with anterior	10 (3.4)	4 (1.4)	6 (2.1)
Between anterior and posterior (in posterior)	10 (3.4)	6 (2.1)	4 (1.4)
Between anterior and posterior (in anterior)	6 (2.0)	4 (1.4)	2 (0.7)
Inside the medial segment (middle lobe)	1 (0.3)	1 (0.3)	–
Between medial lateral (middle lobe)	15 (5.2)	15 (5.2)	–
Between ABS and LBS	56 (19.4)	33 (11.5)	23 (8.0)
Between LBS and PBS	19 (6.59)	11 (3.8)	8 (2.8)
Between MBS and PBS	42 (14.58)	18 (6.3)	24 (8.3)
Between MBS and ABS	128 (44.4)	93 (32.3)	35 (12.2)
Intralingual segment	8 (2.8)	–	8 (2.8)

*n* Number, *%* frequency, *ABS* anterobasal segment, *LBS* laterobasal segment, *MBS* mediobasal segment, *PBS* posterobasal segment

The most frequent accessory fissure variation in the right lung was between the mediobasal and anterobasal segments, occurring in 34.9% of female and 30.2% of male patients. In the left lung, this variation was also the most common, observed in 11.1% of female and 13.1% of male patients. Except for one variation type, no significant gender differences were noted in fissure variations in either lung side (*p* > 0.05). The variation between the anterobasal and laterobasal segments was significantly more common in male (14.8%) than in female patients (7.1%) in the right lung (*p* < 0.05; Table 3).

**Table 3:** Anatomical variations in fissures according to gender

Accessory fissures	Right lung		Left lung	
	Female, *n* (%)	Male, *n* (%)	Female, *n* (%)	Male, *n* (%)
Azygos	3 (2.4)	2 (1.2)	–	–
Apical with posterior (superior)	6 (4.8)	3 (1.9)	3(2.4)	3 (1.9)
Between superior and basal (inferior) superior oblique fissure	19 (15.1)	17 (10.5)	10(7.9)	10(6.2)
Medial basal boundary	0 (0)	0(0)	0 (0)	0(0)
Apical with anterior	2 (1.2)	2 (1.2)	2(1.6)	4(2.5)
Between anterior and posterior (in posterior)	2 (1.6)	4 (2.5)	1(0.8)	3(1.9)
Between anterior and posterior (in anterior)	2 (1.6)	2 (1.2)	0 (0)	2 (1.2)
Within the medial segment (middle lobe)	0 (0)	1 (0.6)	–	–
Between medial lateral (middle lobe)	7 (5.6)	8 (4.9)	–	–
Between ABS and LBS	9 (7.1)	24 (14.8)	7(5.6)	16(9.9)
Between LBS and PBS	3 (2.4)	8 (4.9)	5(4.0)	3(1.9)
Between MBS and PBS	8(6.3)	10(6.2)	8(6.3)	16(9.9)
Between MBS and ABS	44(34.9)	49(30.2)	14(11.1)	21(13.0)
Intralingual segment	–	–	3(2.4)	5(3.1)

Test: ×2, *p* < 0.05

*MBS* mediobasal segment, *PBS* posterobasal segment, *ABS* anterobasal segment, *LBS* laterobasal segment

## Discussion

Pulmonary fissure variations are very important for clinicians and surgeons. Since bronchopulmonary segments are surgical units, they can be resected to preserve adjacent lung tissue. Many pathologies such as bronchopneumonia, mycobacterial infections, tumors, aspiration, pulmonary infarction, and sequestration show segmental involvement, and thus mastery of segmental anatomy is clinically important [12, 13]. Utilizing narrow collimation, high-spatial-resolution reconstruction algorithms, and a small field of view (FOV) significantly improves the spatial resolution of MDCT images, facilitating the identification of fissures [14]. Our study, in contrast to many cadaver lung studies in the literature, aimed to ascertain the prevalence of major, minor, and accessory fissures in a healthy population, thereby aiding clinicians in surgical planning. Furthermore, this research is distinct from other work, since it is grounded in the comprehensive fissure classification by Lange and Walsh [11] as opposed to the widely used Craig–Walker classification ([15–21]; Table 4). The Lange–Walsh classification is more comprehensive than the Craig–Walker classification. It allows for a more detailed and thorough analysis of fissure variations. The Craig–Walker classification poses challenges in radiological decision-making, especially when determining whether a feature is below or above the 50% threshold. Given the difficulty in making such assessments based on CT images, particularly in cases hovering around the 50% mark, and the practical challenges of employing this classification in reporting, we opted for Lange and Walsh’s classification system.

**Table 4:** Comparison of different studies indicating the variations in major and minor fissures

				Right lung							Left lung				
	Year	Total sample	Materials	Oblique fissure			Horizontal fissure			Accessory fissures	Oblique fissure			Horizontal fissure (minor fissure)	Accessory fissures
				Complete	Incomplete	Absent	Complete	Incomplete	Absent		Complete	Incomplete	Absent		
Our study	–	–	MDCT	67.7	31.3	1	62.2	35.1	2.8	81.59	70.1	27.1	2.8	18.4	47.22
Mpolekeng et al.	2022	39	Cadaver	5.1	69.2	2.6	20.5	59.0	0	–	38.46	38.46	2.6	–	–
Joshi et al.	2022	70	Cadaver	66.5	22	12.5	64.5	12.5	25	–	69	21	10	–	–
Mutua et al.	2021	48	Cadaver	90.69	2.3	0	72.03	11.6	11.6	2.3	81.39	2.3	0	–	16.3
Sudikshya	2018	50	Cadaver	69.57	30.43	0	52.18	34.78	13.04	26.07	48.15	51.85	0	26.92	3.70
Quadros et al.	2018	40	–	94.44	5.55	0	63.88	25	11.11	13.88	97.5	2.5	0	17.5	5
Lakshmi et al.	2018	60	Cadaver	86.67	13.33	–	66.67	30	3.33	4	71.67	28.33	–	–	–
Gopalakrishna et al.	2017	100	Cadaver	82	16	0	74	20	6	6	72	0	18	–	4
Shivleela et al.	2017	84	Cadaver	16	63	1	11	63	26	–	21	70	9	–	–
Dhanalakshmi et al.	2016	50	Cadaver	68	32	0	30	52	18	8	62	38	0	2	4
Thapa et al.	2016	40	Cadaver	70	30	0	30	50	20	–	60	25	15	–	–
Magadum et al.	2015	40	Cadaver	30	60	0	35	52.5	12.5	7.5	84.94	15.06	0	7.5	8
Varalakshmi et al.	2014	64	Cadaver	83.3	16.7	0	60	30	10	7.9	68	29	3	37.5	0
Ambali et al.	2014	100	Cadaver	72	14	4	64	28	8	38	78	18	4	–	32
George et al.	2014	65R, 73L	Cadaver	96.93	3.07	0	61.55	35.38	3.07	–	84.94	15.06	0	2.73	–
Nene et al.	2010	50	Cadaver	92	6	2	78	8	14	18	88	12	0	–	50
Meenakshi et al.	2004	30	Cadaver	63.4	36.6	–	19.8	63.3	16.6	–	53.4	46.6	–	–	–

Grading fissures is important for facilitating an easy approach in the surgical procedure and for preventing postoperative hemorrhage and complications. An incomplete major fissure can create an unusual fluid trail within the fissure, leading to potential misinterpretation by physicians [22]. Incomplete or absent major and minor fissures can also modify the intrapulmonary spread of diseases. For example, pneumonia confined within the lobe boundaries may be contained by a complete and normal fissure. Conversely, in patients with complete fissures, pneumonia can extend to adjacent lobes via parenchymal continuity [23]. Similar mechanisms can explain atypical lobe involvement in lung carcinoma. Heřmanová and colleagues identified incomplete oblique fissures in 24% of left and 35% of right lungs, predominantly in the parahilar region [5]. They also found 74% of cases exhibited an incomplete horizontal fissure. Lukose et al. reported that 21% of horizontal fissures were incomplete and 10.5% were complete, and noted a 21% incidence of incomplete oblique fissures in left lungs [24]. Nene et al. identified right horizontal fissures as 8% incomplete and 14% absent, and left oblique fissures as 12% incomplete [25]. Our study revealed that 31.3% of oblique fissures were incomplete on the right and 27.1% were incomplete on the left side. Compared to the studies by Lukose et al. [24], Meenakshi et al. [17], and Bergman and Afifi [26], our research observed higher variation rates in all parameters. Notably, oblique fissures were absent in 1% of right and 2.8% of left cases, a finding not previously reported. Precise segmental localization is crucial for many disease lesions, making knowledge of a frequently occurring pulmonary fissure like left horizontal fissure extremely important clinically. The existence of such a fissure can significantly influence preoperative planning and strategies for segmental resection or pulmonary lobectomy. The incomplete nature of major and minor fissures could contribute to postoperative air leakage. These incomplete fissures often act as barriers against infection spread, forming sharply defined pneumonia that may be erroneously interpreted as atelectasis or consolidation.

In our research, accessory fissures were identified in 81.59% of right lungs and 47.22% of left lungs. Another study suggested that accessory fissures could be present in 8–25% of cases [30]. The literature commonly cites azygos, superior, and inferior accessory fissures as well as left horizontal fissures as the most frequent accessory fissures [31, 32]. Heřmanová et al. reported an equal prevalence of accessory fissures on both sides (16%), with accessory horizontal fissures (8%) being most common on the right and superior accessory fissures (7.2%) on the left [5]. They also observed, as in our study, that the occurrence of accessory fissures showed no correlation with gender or side. Our findings suggest that accessory fissures may be significantly more prevalent than previously assumed. Lung fissures play a dual role: They facilitate the expansion of lung tissue and act as reliable anatomical landmarks [33]. Accessory fissures, when present in atypical locations within the lungs, can lead to the formation of abnormal lobes ventilated by normal bronchi [34]. In patients with endobronchial lesions, accessory fissures can alter the typical pattern of lung collapse, presenting an unusual appearance that complicates the diagnosis of both the lesion and its spread [17].

In our study, the most frequently encountered fissure was the inferior accessory fissure. Inferior accessory fissures, encircling the medial basal segment (S7) of the inferior lobe, separate this segment from the remainder of the lower lobe. Consequently, this segment is referred to as the inferior accessory, cardiac, retrocardiac, or infracardiac lobe [25]. Previous research has identified an accessory structure that separates the anterobasal segment from the mediobasal and posterobasal segments, commonly referred to as the *inferior accessory fissure*, which is often incomplete [3, 10]. However, in contrast to Lange et al., we treated the inferior accessory fissure as two distinct accessory fissures. Our findings revealed that the fissure between the mediobasal and posterobasal segments (MBS-PBS) was present in 6.3% of cases, and the fissure between the mediobasal and anterobasal segments (MBS-ABS), in 34.9%. Studies by Yıldız et al. [3], who considered these two fissures as one (inferior accessory fissure), reported its occurrence to be 12%, while Arıyürek et al. reported a prevalence of 21%. Autopsy studies have suggested that inferior accessory fissures are found in 40–50% of cases [17, 33].

In our research, the second most frequently observed accessory fissure was found between the superior and basal segments of the lower lobe, often termed the *superior accessory fissure* (19.4%), along with the fissure between the anterobasal and laterobasal segments (ABS-LBS; 19.4%). We noted a higher prevalence of these fissures on the right side, although the difference did not reach statistical significance. Nene et al. [25] have reported the presence of superior accessory fissures in 4% of right lungs, with no occurrences on the left side. This fissure delineates the superior segment of the lower lobe from the basal segments, and when present, this segment is sometimes referred to as the *posterior* or *dorsal lobe*. Typically, this fissure is situated at or slightly below the level of the horizontal fissure [30]. Various studies suggest that the prevalence of the superior accessory fissure ranges from 5% to 20% [36–39]. A comprehensive understanding of fissure variations is crucial for accurate assessment of the segmental localization of lung lesions and the progression of lung diseases. It also assists in differentiating accessory fissures from normal anatomical and pathological structures. Familiarity with these variations is essential for modifying preoperative strategies in procedures like pulmonary lobectomy and thoracoscopic segmentectomy. In addition, automated reconstruction of pulmonary segments helps to select the appropriate resection method in the surgical treatment of lung cancer.

## Conclusion

Our review of the literature indicated a notable gap in information regarding certain accessory fissures. However, as demonstrated in our study, these accessory fissures tend to occur more frequently than is widely recognized. Accessory fissures in patients with endobronchial lesions might alter the usual pattern of lung collapse and pose challenges in diagnosing a lesion and its extent. Often these accessory fissures act as a barrier to the spread of infection, creating a sharply marginated pneumonia, which can wrongly be interpreted as atelectasis or consolidation. An anomalous fissure can be mistaken for a lung lesion or an atypical appearance of pleural effusion.

## Data Availability

The datasets used and/or analyzed during the current study are available from the corresponding author on reasonable request.

## Declarations

All procedures performed in studies involving human participants or on human tissue were in accordance with the ethical standards of the institutional and/or national research committee and with the 1975 Helsinki declaration and its later amendments or comparable ethical standards. This retrospective cross-sectional study was conducted after approval was obtained from the clinical research ethics committee of Ondokuz Mayis University (OMUKAEK NO: 2021/494). Informed consent was obtained from all individual participants included in the study.

The supplement containing this article is not sponsored by industry.
